# Household-level sanitation in Ethiopia and its influencing factors: a systematic review

**DOI:** 10.1186/s12889-022-13822-5

**Published:** 2022-07-29

**Authors:** Josef Novotný, Biruk Getachew Mamo

**Affiliations:** Department of Social Geography and Regional Development, Faculty of Science, Charles University, Prague, 12800 Czechia

**Keywords:** Environmental health, Ethiopia, Latrine adoption, Sanitation, Systematic review

## Abstract

**Background:**

Within the past two decades, Ethiopia has achieved one of the fastest reductions of open defecation worldwide. This change can be attributed to the implementation of a national sanitation strategy that focused on facilitating community demand for latrine adoption and use of basic self-constructed latrines but less on other preconditions of hygienic sanitation. Recognition of sanitation by policymakers also catalyzed primary research in this area. As such, the synthesis of the available evidence is both warranted and possible. In this article, we thus decided to assess available primary evidence on the household-level sanitation in Ethiopia and its influencing factors.

**Methods:**

We searched primary studies that present findings on the role of factors influencing household-level sanitation outcomes in Ethiopia. We typologically classified sanitation outcomes analyzed in identified literature and computed pooled estimates for the most prevalent ones (measures of latrine availability and use). We characterized thematic types (themes and sub-themes) of influential sanitation drivers and used network analysis to examine the relational patterns between sanitation outcomes and their influencing factors.

**Findings:**

We identified 37 studies that met our inclusion criteria—all but one published after 2009. The general latrine coverage pooled across 23 studies was 70% (95% CI: 62–77%), the share of improved latrines pooled across 15 studies was 55% (95% CI: 41–68%), and latrine use pooled across 22 studies was 72% (95% CI: 64–79%). Between-study heterogeneity was high, and no time trends were identified. The identified sanitation outcomes were classified into eight types and factors reported to influence these outcomes were classified into 11 broader themes and 43 more specific sub-themes. Factors around the quality of latrines represented the most frequent sub-theme of consequential drivers. We found that the available research focused predominantly on outcomes concerning the initial adoption and use of basic latrines, emulating the main focus of national sanitation strategy. By contrast, research on drivers of the sustainability of sanitation change and, in particular, on the upgrading of latrines, has been rare despite its urgency. There is a high need to redirect the focus of sanitation research in Ethiopia towards understanding these factors on both the demand and supply side.

**Supplementary Information:**

The online version contains supplementary material available at 10.1186/s12889-022-13822-5.

## Introduction

A global sanitation target incorporated into the Sustainable Development Goals (SDGs) aims to achieve access to adequate and equitable sanitation for all and end open defecation (OD) by 2030 [1]. Despite improvements, it is unlikely that the SDGs sanitation target will be met [2]. There are pronounced inequalities in sanitation conditions across the world with the worst situation being in Sub-Saharan Africa. There, only one fifth of the population had access to safely managed sanitation facilities in 2020 [3].

Along with the inclusion into the SDGs, sanitation has been recognized among development and public health priorities in many low- and middle-income countries which have implemented large-scale sanitation programs. Concurrently, sanitation research has also gained momentum. The volume of primary evidence has increased as several systematic reviews were recently published that primarily assessed the effects of sanitation interventions [4–9].

Although informative, it is argued that the focus on interventions’ effectiveness may not be enough to fully understand how complex context-sensitive sanitation conditions evolve [10–13]. Thus far, factors that are potentially important for sanitation outcomes have been viewed through conceptual frameworks such as the IBM-WASH [14] or the RANAS model [15]. They provide comprehensive classifications of theoretically and empirically justified factors and/or mechanisms that are important to consider as potentially important WASH drivers. However, they do not systematically quantify the occurrence of specific consequential drivers (or their thematic types) based on available empirical literature.

With an estimated population of more than 115 million, Ethiopia plays a major role concerning both regional and global trends in sanitation indicators. Between 2000 and 2020, the country recorded the most notable reduction of OD worldwide from 79% to 17% [3, 16]. This pronounced change can largely be attributed to efforts coordinated under the national sanitation strategy [17–19] implemented through the country-wide health extension program [20, 21]. The sanitation strategy adapted Community-Led Total Sanitation (CLTS) as a central approach, so Ethiopia was among the first countries to implement it at scale since 2006 [22]. However, this reduction of OD was achieved predominantly by ensuring access to sanitation facilities which often fail to meet basic hygienic standards. Only 7% of them were classified as safely managed in 2020 [3]. This brings into question the presumed public health effects of toilet adoption in Ethiopia. The low quality and durability of latrines is also a major risk for the return of OD [23]. More recent sanitation policies have recognized these challenges [24, 25], yet their results remain to be seen. Household-level sanitation continues to be a priority of Ethiopian public policy, and the present attempt to synthesize available evidence on its drivers is warranted.

The general aim of our study is to assess published research that analyzes the role of drivers of household-level sanitation outcomes under similar geographical and institutional settings. Therefore, the focus of our study is on a single country, Ethiopia. We conducted systematic search of primary literature that reported research findings on how various factors influence sanitation outcomes at the household level in Ethiopia. Our more specific objectives are (1) to characterize this literature, (2) to examine the evidence concerning the major outcomes analyzed in these studies, (3) to identify factors that influence these outcomes, and (4) to analyze the relationships between the factors and outcomes.

## Methods

Focusing on factors influencing sanitation outcomes, this study represents a specific type of systematic review. It is not based on any registered protocol, but a global review with similar objectives as this study [26] served as a template, particularly with respect to data extraction and presentation.

### Study eligibility and search

Primary studies that analyzed household-level sanitation and its influencing factors in Ethiopia were considered for this review. Importantly, only those studies which analyzed sanitation measures as main study outcomes and provided information on factors of these sanitation-related outcomes were considered. We thus excluded studies that employed sanitation measures solely to examine other outcomes (such as the environmental measures of exposures to pathogens or epidemiological measures of health conditions) if they made no attempts to explain the observed sanitation conditions. We applied no exclusion criteria regarding the type of considered factors. It means that we considered the contextual, psychosocial as well as technology factors in terms of the IBM-WASH classification of factors [14].

No restrictions were applied with respect to the study type and research design. We also searched for studies that examined household-level sanitation conditions in both rural and urban settings and in both interventional and non-interventional settings. We searched for primary research studies published between 2000 and 2021. As previously mentioned, OD was reduced substantially during this period, and the nationwide health extension program has been implemented since 2003 which has been important for addressing sanitation in Ethiopia. Together with the increased emphasis on sanitation in the global strategic frameworks such as SDGs, research on sanitation in Ethiopia increased significantly after 2000 which was also confirmed by our preliminary literature search.

The initial searches for this review were done between February and March 2020 and addressed both literature in peer-reviewed journals and grey literature written in English or Amharic. Additional searches for more recently published studies were conducted at the beginning of October 2021 but focused only on the Web of Science and Scopus databases (compromise between practical constraints and usefulness of our previous searches). The following databases (or search engines) were searched in our initial searches: PubMed, Web of Science, Scopus, Google Scholar, Campbell Collaboration Library, Cochrane Collaboration Library, International Initiative for Impact Evaluation evidence portal, and Addis Ababa University digital library. In addition, the following organizations’ website resources were searched: Africa Development Bank: Water and Sanitation Program, World Bank: Water and Sanitation Program, World Health Organization, United Nations Children’s Fund, International Water and Sanitation Center, Plan International, USAID, UK Department for International Development, One WASH program, and Ethiopian Ministry of Health. The following search string was used where applicable: “(latrine OR toilet OR privy OR child faces OR open defection OR CLTS OR sanitation) AND Ethiopia.” When not applicable, individual keywords or their combinations were searched. In addition, a few potentially relevant papers were also found by screening references in previously identified studies (done for 183 studies that were assessed for eligibility).

The initial searches were conducted by one of the authors (BGM) and monitored by the second reviewer (JN) who was involved in the additional searches for more recent studies. Ambiguities that emerged during the search and selection phase were discussed and sorted out by both scholars. When the initial searches were completed, we eliminated duplicate records and excluded irrelevant studies based on the assessment of titles and abstracts (mostly done by BGM). The full texts of those studies that were found to be potentially relevant were then assessed based on whether they met our inclusion criteria or not (both authors participated in this).

### Data extraction and analysis

The following four general types of data were extracted into predefined extraction forms from studies in our sample. First, characteristics of individual studies such as bibliographic information, geographical focus, design, sample size, and method of data collection were recorded. Second, we extracted information on specific sanitation outcomes that were examined in individual studies and the methodology of their measurement. Third, we extracted information on individual factors influencing sanitation outcomes. More specifically, for relationships that were reported as statistically significant in quantitative studies (*p*-value less than 0.05) or consequential in qualitative studies we recorded factor-outcome relationships and specific details such as their direction, effects size and confidence intervals (if quantitative). We grouped the factors into themes and then broke down into sub-themes. We assessed the representation of these themes and sub-themes by counting the frequency of occurrence of significant/consequential factors pertinent to these themes and sub-themes. The extraction was firstly conducted by one reviewer (BGM) and then checked in detail by a second reviewer (JN).

The data analysis consisted of the following steps which also correspond to the presentation of findings in the ﻿Results section below. First, we considered the feasibility of quantitative summarization of identified outcomes and decided to calculate pooled estimates for three measures of the two most prevalent outcomes in terms of latrine coverage and use. Because of the substantial between-study heterogeneity, we calculated pooled estimates (their confidence intervals) using random-effect models as in [27]. Second, we typologically classified and descriptively characterized identified sanitation outcomes. Third, we thematically classified the identified consequential drivers of these outcomes at two hierarchical levels into themes and sub-themes and assessed their representation. Fourth, we discussed relationships between factors and outcomes with the emphasis on their underlying mechanisms. For example, we inspected whether individual factor-outcome relationships classified into the same themes and sub-themes have identical and expected directions. If not, we looked into the respective studies and confronted their explanations of mechanisms operating beyond these relationships. In this fourth step, we also examined the pattern of these relationships by constructing a network visualization that depicts how particular sub-themes of influential factors interlink different types of sanitation outcomes. The network was constructed using the Cytoscape software [28] based on the edge-weighted spring embedded algorithm with the size of sub-themes considered as weights.

All data generated or analyzed during this study are included in this published article [and its supplementary information files].

## Results

### Final sample of studies

**Fig. 1:**
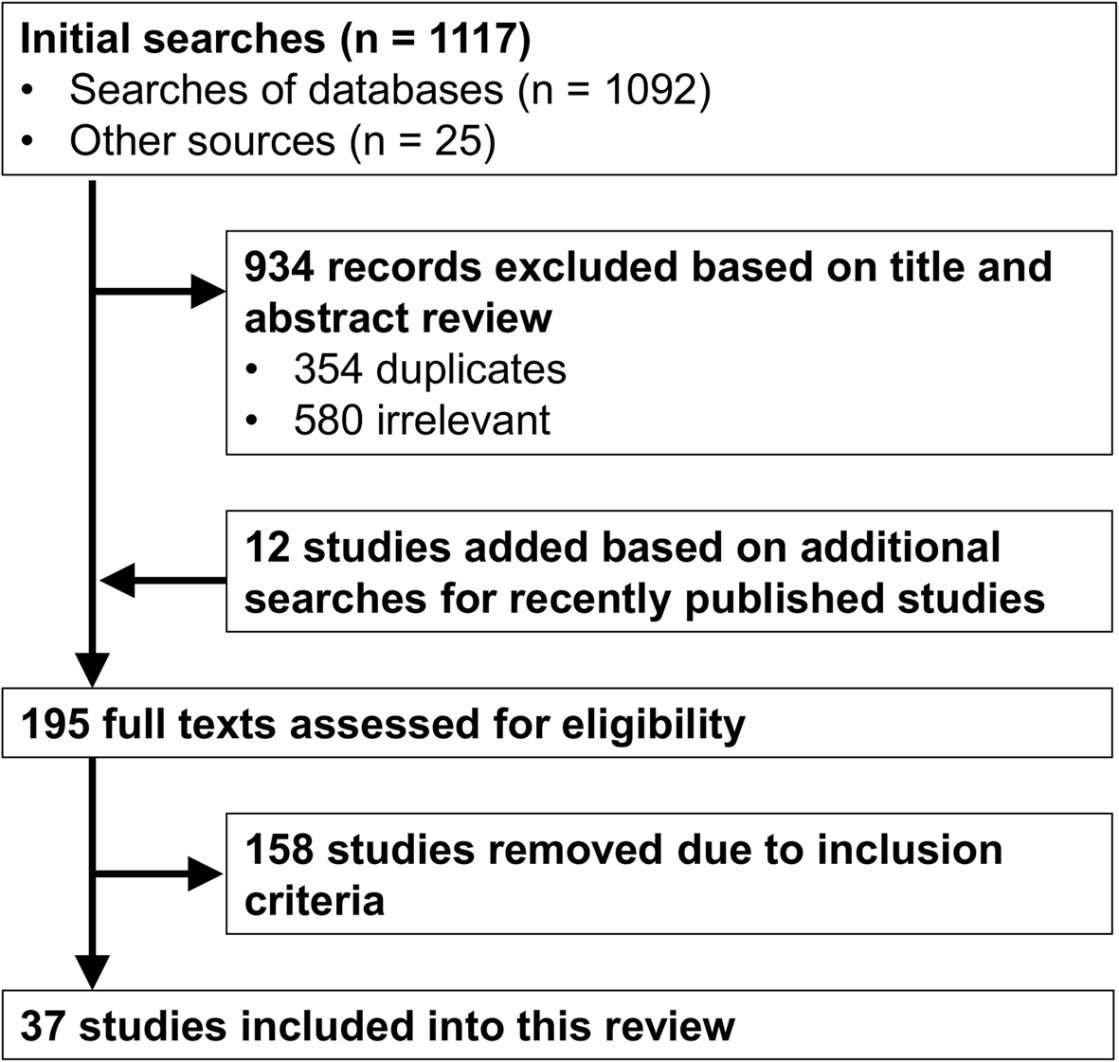
Flow chart of study selection process

After removing duplicate and irrelevant records obtained through our searches, we examined the full texts of 195 studies—37 of which met our inclusion criteria (Fig. 1). Several studies which collected data on household-level sanitation in Ethiopia were excluded due to the eligibility criteria. They mostly analyzed sanitation measures as explanatory variables for examining other outcomes but did not report on factors influencing these sanitation measures. Some examples are [29–31]. Studies that were included in our sample are listed in Supplementary Table S﻿1 with their descriptive characteristics. Although we searched for literature published since 2000, the earliest study that we identified was [32] based on data collected in 2004. The majority of studies in our sample were published in recent years (70% after 2015).

Four studies provided comparisons of samples collected in more than one regional state, while the remaining 33 studies used data from a single region. Most of the studies were conducted in the following four regions: Amhara (16 studies), Southern Nations Nationalities and Peoples’ Region (SNNPR) (11), Tigray (10), and Oromia (9). Only three studies contained data collected in additional regions [33–35]. This can be partially explained by differences in the size of regional states, but also by their centrality (both locational and political) and their history with sanitation interventions (the first campaign for universal sanitation implemented in SNNPR since 2003 [36]. Only four studies focused on urban or semi-urban areas [35, 37–39]. Although there are different challenges posed by urban and rural sanitation, we decided to keep these four studies in the sample because they were comparable to other research regarding technology and observed sanitation patterns (decentralized sanitation, simple pit latrines, either with or without slab platforms). We searched for research works written in English and Amharic as mentioned in the Methods section. However, we did not identify any study in Amharic that would qualify for the full-texts assessment of eligibility criteria (as in Fig. 1) and only papers written in English qualified for the sample. Except for one organizational report [34], all studies were journal articles. Regarding the design, studies based on cross-sectional data analyzed quantitatively clearly dominate the sample (33 studies). A few of them (5) collected qualitative data simultaneously, while two other studies solely utilized qualitative data [40, 41]. Only three studies collected data that allowed to directly examine changes in sanitation outcomes over time [42–44] and, exceptionally, the drivers influencing these changes in sanitation outcomes ([44] represents a sole example of such findings).

The majority of studies in our sample (24 of 37) did not focus on any specific intervention, though the implementation of a national sanitation policy was commonly acknowledged as an important motivation and implicit contextual feature. The remaining research included in our sample was more or less explicitly designed to analyze specific types of sanitation interventions such as the WASH components of the trachoma control program implemented in the 2000s [32, 42, 45] or a specific locally implemented promotion of composting “arborloo” latrines [46] or various types of Ethiopian adaptations of CLTS (examined in nine studies).

### Quantitative characterization of most frequent sanitation outcomes

Although multiple different types of sanitation outcomes were identified, measures of latrine use and availability were the most frequently represented (see next section). We decided to attempt the quantitative summarization of results on three measures related to these most frequent outcomes across studies (Table 1). More specifically, we were able to extract information on latrine availability (coverage) from 23 studies, on the improved latrine availability from 15 studies, and on latrine use from 22 studies (Supplementary Table S﻿2). As shown in Table 1, on average around 70% of households had access to a latrine, 72% of latrine-owning households used them, and 55% of latrines were improved latrines, meaning that they contained at least basic solid slabs assumed to separate excreta from human contact. Figures extracted from individual studies were determined based on data collected between 2004 and 2019 in different parts of Ethiopia. Nevertheless, we did not identify any notable time trends or geographic variations regarding the pooled measures of latrine use and prevalence of improved latrines. For latrine availability, 10 studies based on surveys conducted between 2013 and 2015 reported generally higher levels of latrine availability compared to both earlier and more recent surveys, but we found that this was attributable to geographical variations. Studies conducted in South-Western Ethiopia (Oromia and particularly SNNPR) revealed higher latrine availability rates (with an average of 77%, based on 13 studies) compared to those from the North-West (Tigray and Amhara; 12 studies with an average of 58%).

**Table 1:** Pooled estimates of basic indicators of latrine coverage and use

	Latrine availability (any latrine)	Improved latrines^a^ of all latrines	Latrine use among latrine-owning households
Number of studies	23	15	22
Households covered altogether	19810	11793 (latrine-owning households)	13742 (latrine-owning households)
Range	0.36–0.98	0.16–0.93	0.47–0.97
Pooled average (unweighted)	0.70	0.55	0.72
95% CI for pooled average	0.62–0.77	0.41–0.68	0.64–0.79

^a^Improved latrines are defined as latrines with solid slab platforms

However, the pooled averages should be interpreted with caution because of considerable between-study heterogeneity. The latter is obvious from the wide ranges of values extracted from individual studies, wide confidence intervals (CI), and high *I*^2^ statistics (above 90% for all three outcomes). The heterogeneity stems from variations in study designs and, perhaps even more notably, from contextual differences between studies, including pronounced geographical variations in sanitation conditions across Ethiopia. Moreover, in addition to the between-study heterogeneity, some of the studies also documented considerable within-study heterogeneity in terms of differences in the levels of analyzed outcomes between different sites or regions within their samples [43, 47].

### Classification of sanitation outcomes

We classified specific outcomes that were examined in 37 studies in our sample into the eight types (Table 2). The frequencies of occurrence of these types were assessed based on the number of studies that analyzed them (second column of Table 2) and also based on the number of significant/consequential relationships between identified factors and these outcomes (third column)—as in Supplementary Table S﻿3. Although directly related, the identified outcome types refer to typologically distinct aspects of sanitation conditions or changes in these conditions. The most frequent type of outcome was latrine use analyzed in 24 studies, followed by latrine availability (11 studies), OD (10), and latrine adoption (10). Latrine adoption predetermines latrine availability and, intuitively, both these outcomes are inversely related to OD. However, we realized that the set of studies that examined latrine availability largely differed from the set of studies that analyzed latrine adoption and open defecation. Overall, only three studies reported on drivers of all these four interrelated types of outcomes simultaneously [33, 36, 40].

**Table 2:** Identified types of sanitation outcomes

Broader types of outcomes	Nm. of studies	Nm. of observations^**a**^	Specification of outcomes as examined in individual studies
Latrine use	25	126	Latrine use; consistent latrine use by family members; latrine utilization
Latrine availability	11	43	Latrine availability, latrine ownership, availability of improved latrine
Open defecation (OD)	10	41	OD; reasons for not constructing latrine; reasons for not using latrine; perceived disadvantages of OD
Latrine adoption	10	55	Latrine adoption (generally); latrine construction; reasons for constructing latrine; adoption of arborloo latrine
Sustainability of latrine adoption	8	42	Sustainability of latrine use and latrine quality (longitudinal focus); reasons for abandoning latrine use; re-construction of latrines and reasons thereof; sustained use of arborloo latrines
Satisfaction with latrine use	5	18	Perceived advantages/benefits of latrine and its use; satisfaction with latrine use; reasons for dissatisfaction with latrine
Latrine quality improvement	3	6	Improvement of latrine; Intention to improve latrine; reasons for (not) improving latrine
Sanitation safety	1	5	Composite score based on 11 characteristics of availability, quality, and use of latrines

^a^﻿Number of observations (or links) refers to the number of identified significant/consequential relationships between specific factors and sanitation outcomes as examined in the “Relationships between factors and outcomes” section

Studies that reported on factors influencing latrine availability and adoption were always concerned with access to private latrines but differed in the definition of sanitation facilities (e.g., whether the focus was on the presence of any, solely functional, or improved latrines). Latrine availability represents a necessary but not sufficient condition for latrine use. Nevertheless, several studies in our sample reported on the drivers of latrine use but not on those influencing latrine availability, including nine articles that purposely analyzed latrine use among solely latrine-owning households.

Other types of outcomes shown in Table 2 capture specific aspects around household-level sanitation such as the sustainability of latrine adoption, satisfaction with latrine use, or quality improvement of sanitation facilities. In addition, one study focused on the composite score of sanitation safety based on multiple characteristics of household-level sanitation situations [48]. Most of the identified types of sanitation outcomes can be classified by considering their position in the sanitation ladder in terms of the processual chain towards a safer sanitation environment with the following sequence: OD → latrine adoption → latrine availability → latrine use → satisfaction with latrine use → latrine quality improvement → sustainability of latrine adoption. From this perspective, Table 2 documents that available research on household-level sanitation in Ethiopia has disproportionately focused on the initial parts of the process, while studies focusing on the drivers of sustainability of latrine use and quality improvements are comparatively less represented.

We further inspected how latrine use as the most represented outcome was measured in the analyzed papers. The majority of studies measured latrine use based on self-reported information about sanitation behavior obtained from respondents in interviews (19 of 25 studies focused on this outcome). We assume that at least some of them validated information about self-reported behavior by direct observation of respondents’ sanitation facilities, but only one of them [43] explicitly mentioned this in the methodology description. Four studies solely considered observations of the signs of latrine use without relying on self-reported information about sanitation behavior [32, 39, 47, 49] and two studies combined information from interviews with the observations of latrines [50, 51]. In general, however, the measurement of latrine use was poorly described, and in a few cases, the definitions of latrine use provided in the methodology descriptions diverged from what was actually reported in the results [37, 52].

### Typology of influencing factors

The analysis of 37 studies included in this review yielded 336 links between factors influencing outcomes described above (Supplementary Table S﻿3). These are factors that were reported to be the statistically significant correlates of household-level sanitation outcomes in the analyzed quantitative studies or consequential drivers in the examined qualitative studies. We classified these factors at two levels. First, we followed the typology proposed by [26] and classified the identified factors into 11 broader themes. Second, factors within each theme were further divided into more specific sub-themes (43 in total). Both levels of classification are presented in Table 3. Socioeconomic factors were identified in the highest number of studies (in 26 of 37), while factors related to sanitation infrastructure were most frequently reported (16% of 336 identified links). In addition to these two themes, demographic characteristics were also reported in more than half of the analyzed studies as correlates of sanitation outcomes and factors relating to privacy, safety, and/or convenience of sanitation practices accounted for more than 10% of identified links.

**Table 3:** Typology of identified consequential factors (ordered by the number of studies that reported presented themes and sub-themes of factors)

Broader thematic types of factors (***S*** = number of studies; ***N*** = number of occurrences)	More specific sub-themes of factors (number of occurrences)
Socioeconomic factors (*S* = 26; *N* = 44)	Income or wealth (15); general education (11); cost of toilet or its perception (9); agricultural occupation (6); govt employee (3)
Sanitation infrastructure, maintenance, supply, access to materials or manpower (*S* = 24; *N* = 54)	Acceptable quality of latrine (32); availability of material (7); lack of (skilled) manpower (7); need of latrine maintenance (4); unavailability or poor quality of public latrines (4)
Demographic characteristics (*S* = 21; *N* = 36)	Household size (12); children in family (11); female head of household (7); age (4); presence of women (2)
Health and/or cleanliness (*S* = 18; *N* = 36)	Health-related expectations (14); latrine cleanliness (11); cleanliness of environment (7); attract flies (3); experienced health problems (1)
Spatial and environmental factors (*S* = 17; *N* = 31)	Location (centrality, accessibility etc.) (6); lack of space for latrine construction (6); soil, bedrock, terrain suitable for latrine (5); distance of latrine from house (4); climate constraints (floods, rains etc.) (4); access and utilization of water (3); Enough space for OD (3)
Privacy, safety, convenience (*S* = 14; *N* = 38)	Safety (12); privacy (11); convenience (8); smell from latrine (2); smell from OD (4)
Institutional support and/or pressure (*S*= 11; *N* = 29)	Institutional support (26); institutional pressure, command, sanctions (3)
Social pressure, networks, and learning (*S* = 11; *N* = 30)	Social networks, social learning (14); social pressure (11); prestige, status (5)
Hygiene and sanitation knowledge, experience, habits (*S* = 8; *N* = 31)	Recognition of hygiene and sanitation advantages (14); experience with latrine (7); knowledge of CLTSH and its acceptance (6); feces as fertilizer (4)
Satisfied, other priorities (*S* = 4; *N* = 4)	Satisfied with current practice (4)
Cultural factors (bylaws, taboos etc.) (*S* = 2; *N* = 5)	Distinct gender-related cultural norms (3) and other cultural norms (2)

S_TOTAL_ = 37; N_TOTAL_ = 336

Of the more specific sub-themes, factors related to the quality of latrines and institutional support were, by far, the most frequently represented, accounting for 10% and 8% of the identified links, respectively. We acknowledged considerable heterogeneity of measures used to characterize these most represented sub-themes of factors. For example, the following, directly observable parameters were used to measure latrine quality: availability, quality, and material of slab platforms; presence and condition of latrine superstructure; availability of handwash facilities and soaps; whether latrine looks maintained; the presence of doors; whether latrine was worn out; whether (ir)regular shape and structure of latrine; whether squat-hole covered; and easiness of latrine construction.

Similarly, several measures were used for capturing institutional support such as: whether sanitation-related information was received by respondents or their households from health workers and/or volunteers, frequency of supervision by health workers, whether representatives of households participated in organized mobilization activities, whether and how CLTS/CLTSH was implemented in the community, and whether health office/post is available. Unlike the measures of institutional support, the role of commands, pressures, or sanctions commonly used to induce sanitation change in Ethiopia has been considerably less studied.

We tried to compare the distribution of factors uncovered in this article for Ethiopia with those reported in the global review by [26]. We found that factors related to hygiene and sanitation knowledge, demographic characteristics, institutional support, and quality of sanitation infrastructure were comparatively more represented in the present sample of studies focused on household-level sanitation in Ethiopia. The relative occurrences of the two most represented sub-themes of factors (acceptable quality of latrines and institutional support) were also considerably higher for our Ethiopian sample. By contrast, factors capturing privacy, safety, and/or convenience of sanitation practices was a relatively less represented type in our Ethiopian sample compared to the global set of studies analyzed in [26].

In addition to the main classification presented above, we also categorized factors into three general types proposed by the IBM-WASH model [14]. We found that according to the IBM-WASH model definitions 34% of factors identified in our review can be considered as contextual factors, 36% as psychosocial factors, and 30% as technology factors (last column in Supplementary Table S﻿3). The share of the latter category was two times higher than in the global review by [26] in which technology drivers accounted for only 15% of all identified factors.

### Relationships between factors and outcomes

As already indicated, we identified 336 links between specific factors and sanitation outcomes. Hereafter, these links are referred to as observations and they are listed in Supplementary Table S﻿3 with their specifications. More than half of them (193) can be denoted as descriptive findings established as either binary statistical associations or based on qualitative data. The remaining 143 (43%) observations were quantitatively determined using multivariate analytical techniques. They were extracted from 29 studies (of total 37), and in all cases, they were estimates obtained through multivariate regressions, mostly binary logistic regression models, with the effects measured mostly by the adjusted odds ratios. We abandoned an initial plan to quantify pooled effects for the most frequently represented types of observations as we realized that measures used in individual studies for capturing both factors and outcomes are very heterogeneous, and the same holds for the specifications of the underlying regression models.

**Fig. 2:**
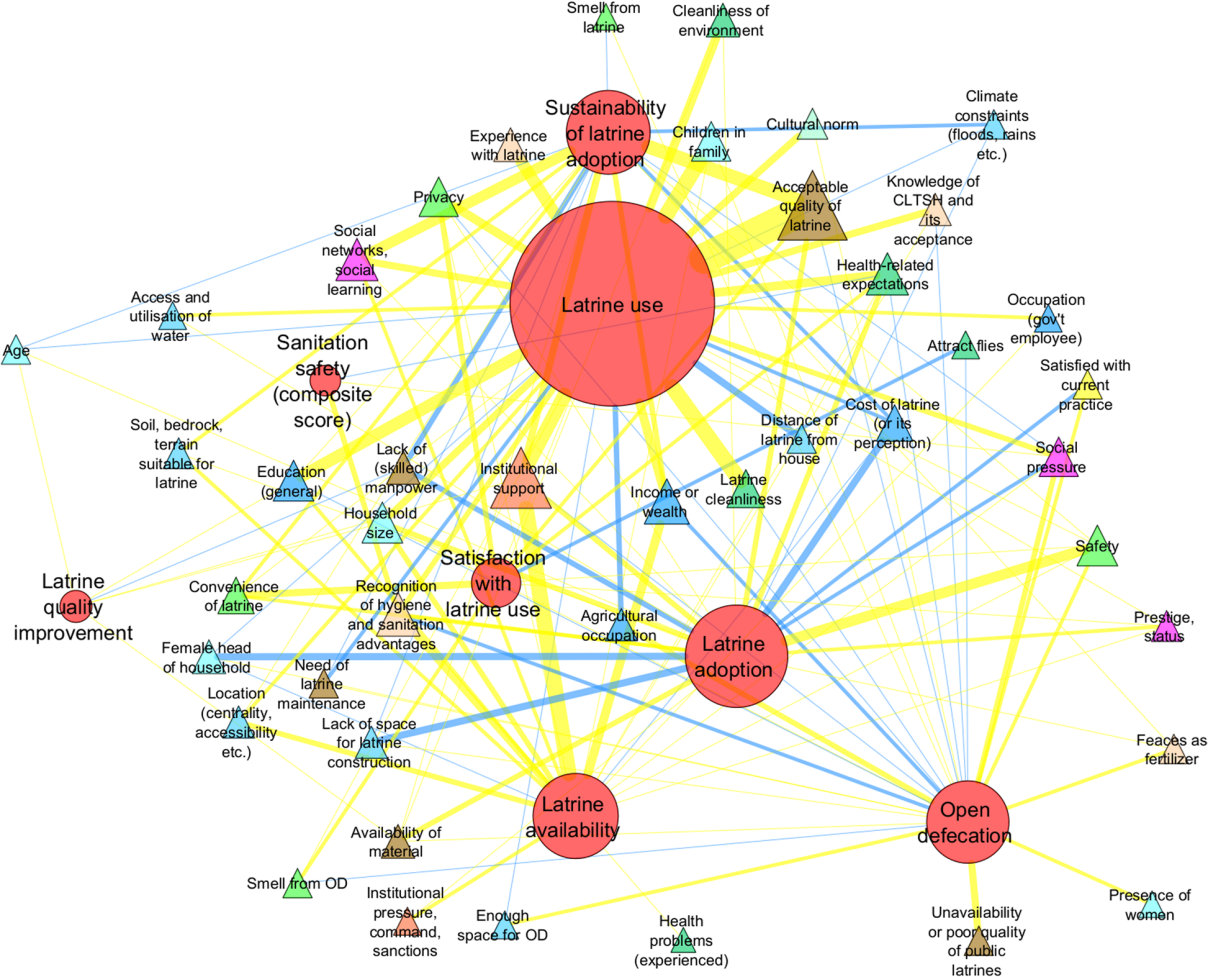
Factors influencing sanitation outcomes in Ethiopia: a network visualization. The circular nodes depict 8 types of sanitation outcomes identified in this review. The triangle nodes show the 43 sub-themes of factors as in the right column of Table 3 and their colors distinguish their 11 broader themes as in the left column of Table 3. Edges represent identified relationships between factors and outcomes (i.e., observations). Positions of circular nodes and their mutual distances in the network indicate the extent to which their respective influencing factors overlap. Edge width and node size indicate differences in the frequency of observations. Yellow edges represent the prevalence of positive relationships and blue edges the prevalence of negative relationships. Network visualization was obtained based on the edge-weighted spring embedded algorithm. Positions of a few triangle nodes were slightly adjusted to prevent overlaps between the labels with no effect on the interpretation

We thus adopted the approach used in [26] and examined the patterns of observations using network analysis (Fig. 2). In addition, Table 4 shows the frequency of identified observations between individual types of sanitation outcomes and themes of factors and Supplementary Tables S﻿4, S﻿5, and S﻿6 provides additional tabular depictions of the distributions of identified relationships. The tables may be easier to read and useful for inspecting the role of specific themes and sub-themes of factors for specific types of sanitation outcomes. However, the network plot in Fig. 2 provides an additional information about the aggregated patterns of identified relationships.

**Table 4:** Number of identified relationships between factors aggregated by themes and sanitation outcomes aggregated by types

	Latrine adoption	Latrine availability	Latrine quality improvement	Latrine use	Open defecation	Sanitation safety (composite score)	Satisfaction with latrine use	Sustainability of latrine adoption	Total
Socioeconomic factors	4	9	1	18	6	0	0	6	**44**
Sanitation infrastructure, maintenance, supply, access to materials or manpower	9	0	2	21	7	0	2	13	**54**
Demographic characteristics	8	5	2	15	5	0	0	1	**36**
Health and/or cleanliness	4	2	0	19	3	1	5	0	**34**
Spatial and environmental factors	7	5	0	10	3	0	0	6	**31**
Privacy, safety, convenience	9	2	0	8	5	0	10	4	**38**
Institutional support and/or pressure	4	11	1	8	1	0	0	4	**29**
Social pressure, networks, and learning	5	4	0	9	3	1	1	7	**30**
Hygiene and sanitation knowledge, experience, habits	3	5	0	14	5	3	0	1	**31**
Satisfied, other priorities	2	0	0	0	2	0	0	0	**4**
Cultural factors (bylaws, taboos, etc.)	0	0	0	4	1	0	0	0	**5**
**Total**	**55**	**43**	**6**	**126**	**41**	**5**	**18**	**42**	**336**

The pattern visualized in Fig. 2 is intuitively interpretable and, once again, can be related to the anticipated positions of particular outcomes in the sanitation ladder. The three types of outcomes capturing the initial part of sanitation change—i.e., OD, latrine adoption, and latrine availability—are located in the bottom part near one another. Latrine use, as the most represented type of outcome, occupies the upper part together with the sustainability of latrine adoption. It suggests that these two types of outcomes are influenced by a similar set of drivers. Improvement of latrine quality, as a less studied outcome type, is located away from other outcomes in the peripheral part of the network.

Positions of triangle nodes that denote factor sub-themes can be interpreted analogously. For example, the most represented sub-theme is the acceptable quality of latrine (32 observations) located in the upper part of Fig. 2. It implies that parameters around the quality of latrines are relatively more consequential for latrine use and sustainability compared to open defecation and latrine availability (which are outcomes at the initial part of the sanitation ladder). This is less true for institutional support which is the second most frequent sub-theme of identified sanitation drivers (26 observations). This node occupies a more central position in the network (based on both visual inspection and measures of network centrality). It implies that institutional support is important for outcomes at various parts of the sanitation ladder.

Different colors of edges in Fig. 2 symbolize the prevalence of positive (yellow edges) or negative (blue edges) relationships concerning how factors from a sub-theme influence an outcome. The directions of relationships were homogeneous and expectable for the majority of factor sub-themes. In some cases, however, factors within the same sub-theme affected sanitation outcomes contrarily. For example, age was negatively related to latrine use in [53] and to the sustainability of latrine adoption according to [40]. Both these studies pointed out differences between elderly households and the rest of the respondents. By contrast, [46] identified a positive relationship between age and latrine adoption, but this finding applies specifically to the age of female heads of households and the adoption of arborloo latrines. Another study [54] also reported a positive relationship between age and latrine quality improvement, while attributing it to differences between very young (less than 25 years) and elderly (over 70) households. The differential effects of age variables may be thus explained by distinct definitions of outcomes, age measures, but possibly also distinct mechanisms operating behind age variables. For example, [40] referred to the lack of capacity of elderly people for adopting toilets, while [54] mentioned higher motivation of elderly people to choose improved latrines because of their physical concerns. Similarly, differential effects were identified for the presence and age of children in households. Studies that considered the presence of children under five years of age reported negative effects of this variable on latrine use [51, 55] and its positive effect on OD [56]. This can be compared to results from a few studies which focused on the presence of school-aged children or children of any age and reported positive relationships with latrine availability and use [39, 47, 50–52, 57–59].

Several of the relationships identified in our sample of studies were related to women, while we distinguish three types of these findings. First, in several studies, female-headed households, which are prevalent across rural Ethiopia, revealed comparatively worse sanitation outcomes [36, 41, 45, 47]. These findings were mainly explained by referring to a generally disadvantageous situation of female-headed households and their lack of capacity to adopt toilets. Second, women were found to practice OD comparatively more than males, reportedly due to the perceived inconvenience and fear of latrine use [41, 47] and women’s responsibility for small children [56]. Third, the literature revealed contradictory findings concerning the gendered socio-cultural norms around sanitation in Ethiopia. It was argued that being seen practicing OD is extremely shameful for women in rural Ethiopia so they are strongly influenced to use latrines consistently [40]. By contrast, [41] mentioned that the unacceptability to share the same latrine with in-laws dissuades married women from latrine use.

Socio-cultural norms partly overlap with other factors classified in Fig. 2 such as social pressure and social networks and learning. In this respect, the identified evidence showed that peer pressures and learning can positively affect latrine adoption, availability, and use, but they can also have opposite effects and inhibit latrine use, adoption, and its sustainability [33, 47, 54, 60].

Convenience, comfort, and safety were reported among motivations for toilet adoption and use. At the same time, safety and inconveniences related to smell, uncleanliness, and the presence of flies were identified as factors discouraging people from consistent latrine use and bolstering OD. Once again, the generally low quality of latrines in rural Ethiopia plays a role in this.

Several studies revealed that expectations that the adoption and use of toilets will prevent diseases and reduce medical expenses are consequential drivers of the examined sanitation outcomes [33, 36, 40, 42, 61]. Actually experienced health conditions were nevertheless rarely reported as significant factors influencing sanitation outcomes. This may be because sanitation measures are mostly examined as determinants of human health without accounting for the reverse relationship. Another explanation may nevertheless be that the actual health impacts of toilet adoption in Ethiopia remain uncertain [62, 63].

## Discussion

We identified 37 studies published between 2010 and 2021 that reported findings on how specific sanitation drivers influence various household-level sanitation outcomes in Ethiopia. Although it does not seem to be a particularly large sample for a priority topic in a country with a population of more than 110 million, it means a notable increase in the research focus on this topic. The majority of studies in our sample were published in recent years, mostly after 2015.This indicates an association between the research focus and political recognition and implementation of sanitation policy in Ethiopia.

Let us repeat that we excluded several studies that also collected data on household-level sanitation in Ethiopia but examined sanitation measures as correlates of other outcomes (such as health outcomes) without attempting to explain observed sanitation conditions. These excluded studies mostly address the more upstream parts of the anticipated chain between environmental sanitation and human well-being. Similarly, we observed that the majority of literature in our sample did not explicitly analyze any specific sanitation intervention, while some studies that focused on impacts of sanitation intervention did not meet eligibility criteria and were excluded. These excluded studies are typically designed to isolate causal impacts of sanitation measures and/or interventions (arguably it is the most common focus of sanitation research published in high ranked journals) without paying much attention to factors influencing examined sanitation measures.

The literature considered for this review is different. Although mostly based on cross-sectional data and thus weaker in the power of establishing cause-and-effect relationships, it is no less important as it facilitates understanding of drivers and conditions that explain intermediate (sanitation) outcomes and operates alongside or independently of interventions (i.e., role of “other” factors). As argued elsewhere, engagement with the type of evidence analyzed in this review challenges a common assumption inherent to the logic of counterfactual (quasi)experimental studies that the other factors represent something that should primarily be controlled for.

Despite the high between-study heterogeneity, we tried to quantitatively summarize results on the three most represented measures of latrine use and availability. The pooled average across 23 studies indicated the general latrine coverage of 70% with the 95% CI of 62–77%. The estimated share of households having improved latrines (of all households with latrines) pooled across 15 studies was 55% with the 95% CI of 41-68%. If combined, these results would imply that only around 39% of Ethiopian households had access to latrines with solid slab platforms, which is considered to be a basic attribute of a hygienic sanitation facility. Our point estimates are not far from the national-level sanitation figures provided for Ethiopia by the WHO/UNICEF Joint Monitoring Programme, which reported the 2017 toilet coverage of 79% with the share improved latrines of 54% [16]. The year of data collection for studies considered for the calculation of the pooled averages spanned the period 2004–2018 and 2007–2019 for latrine coverage and share of improved latrines, respectively (the mean year was 2015 in both cases). We did not identify any notable time trends in the levels of the analyzed indicators. It may imply that there has been little change in the analyzed indicators in Ethiopia since around the year 2015, but the between-study contextual and methodological heterogeneity makes this observation only indicative at most.

In addition, the average of latrine use pooled across 22 studies was 72% with the 95% CI of 64–79%, implying that around 28% of latrine-owning households in Ethiopia do not properly use their sanitation facilities. This rate subsumes both unused and inconsistently used latrines so it may well be compatible with the 16% rate of behavioral slippage from toilet use to OD provided by a recent study by [23]. The measurement of latrine use is nevertheless known to be challenging [64, 65] and our inspection of approaches used for the measurement of this central aspect of sanitation behavior in Ethiopia suggested that the comparability and reliability of findings on this outcome may be problematic.

We identified multiple sanitation outcomes that were examined in the reviewed literature for their influencing factors and classified them into eight broader types. Latrine use was the most frequently analyzed type followed by latrine availability and OD. By contrast, outcomes describing more advanced steps in the processes of change towards a safe and equitable sanitation environment were considerably less often studied. It applies to outcomes such as the sustainability of latrine adoption and the quality/upgrading of latrines that have been rarely examined regarding their underlying drivers even though these issues currently represent the most pressing challenges of sanitation in Ethiopia. These findings mean that the focus of sanitation research in Ethiopia mirrors the nature of Ethiopian sanitation policies (biased towards the focus on the creation of demand for basic latrines) rather than anticipating their gaps and addressing future challenges.

We classified factors that were reported as consequential in the analyzed studies into 11 broader themes and, additionally, into 43 more specific sub-themes. Socioeconomic factors were found in the highest number of studies. The majority of them referred to characteristics such as household income or wealth, education, or occupation. Their frequent associations with household-level sanitation inequalities in Ethiopia is not surprising but underscores the importance of structural change for sanitation in Ethiopia. It reminds us that hygienic and equitable sanitation can hardly be achieved through specific sanitation interventions alone, without general socioeconomic development as a key prerequisite.

The general socioeconomic factors have been interwoven with demographic and cultural sanitation drivers. Perhaps a prime example represents the repeatedly documented adverse sanitation conditions of female-headed households, which signifies how sanitation represents yet another dimension of gendered inequality in Ethiopia. Importantly, a few distinct interpretations of the effects of this factor on sanitation outcomes demonstrate that this aspect of sanitation inequality arises due to an interplay between socioeconomic factors (generally weaker socioeconomic situation of female-headed households, and a lack of capacity or skills to adopt latrines) and cultural factors (prevalence of polygamy, cultural norms around sanitation) and also traditional gender roles (including traditional responsibility of women for water-related issues).

In terms of the frequency of individual observations (relationships between specific factors and outcomes), the most widely reported consequential factors were those around the availability and quality of sanitation infrastructure including access to materials or manpower required for toilet construction and maintenance. Its major sub-theme covers drivers directly related to the acceptable quality of latrines, which itself accounted for nearly 10% of all identified observations. By means of comparison with findings from a global review by [26], we also found that the factors associated with quality of sanitation infrastructure are particularly prominent in Ethiopia. We also took note of the focus on sanitation infrastructure concerned with the presence or cleanliness of the basic pit latrine’s components (slab platform, particular parts of superstructure) or general user assessments (perceived acceptance and convenience). Other consequential aspects such as the supply of adequate infrastructure (including awareness of it at the household level) or alternative technology options and management and reuse of fecal waste (their acceptance at household level) have been rarely studied in Ethiopia.

The second most represented sub-theme of factors were drivers referred to as institutional support. They commonly assessed the presence and delivery of activities of health workers (or other agents involved in sanitation-related mobilization and supervision) such as the frequency of their visits to households or households’ participation in trainings and awareness creation and behavior change campaigns. In studies that were focused on the examination of a specific intervention, these measures often captured the intervention’s delivery. However, they were reported in several other non-interventional studies too. It indicates that differences between interventional and non-interventional studies are blurred due to the implementation of the national sanitation campaign. The latter also constrains the applicability of contra-factual evaluations that may be used for comparing the performance of distinct intervention modalities [43] rather than attributing the net effects of intervention. This remark once again challenges the conventional understanding of what is referred to as the hierarchy of evidence that still uncritically prioritizes experimental studies without adequately considering their limitations and usefulness for practice.

The importance of institutional support for the reduction of OD and initiation of latrine adoption in Ethiopia is clear. Still, we think that the analyzed literature too often conceived and portrayed the measured institutional support as a black box. There were rare exceptions, but generally little can be learned from the analyzed literature regarding the content and nature of institutional pressures. The examined cases of institutional support were mostly presented as positive and unproblematic. The focus on the presence and role of negative pressures and sanctions were considerably scarcer, though it is known that they have been used in Ethiopia. We believe that the prevalent mechanistic approach is unfortunate, particularly considering that implementation fidelity is increasingly recognized as a key problem of the large-scale sanitation interventions in Ethiopia [63].

It may be beyond the scope of our review, but we believe that these comments have wider relevance for the available research on sanitation in Ethiopia. According to our reading of this literature, it tends to be heavily shaped towards the emphasis on routine data collection and descriptive characterization of empirical results. Attempts for a critical interpretation and discussion of findings that may explain sanitation conditions and discuss documented failures seem to be rare. One aspect of this is that the research is often presented as apolitical without an attempt to challenge authorities or touch upon the local politics and power relations involved in the implementation of interventions at the micro-level.

Another notable finding of this review is the confirmation of health-related expectations as consequential motivation for latrine adoption and use in Ethiopia. It was reported more often than non-health motivations such as convenience, privacy, and safety. The prevalent belief that latrine use is good for human health can be contrasted with the uncertain actual health benefits of sanitation change in Ethiopia [62, 63, 66, 67]. It means that there is a relatively good public awareness about the biological plausibility of toilet adoption. Likely, the national sanitation campaign and the community-level persuasion techniques that were used played a role in creating this awareness. However, it simultaneously focused on the adoption of basic sanitation so the conviction about health benefits of latrines coexists with the acceptance of their generally low hygienic standards. It implies that there may be an inadequate perception of what a hygienic toilet is. Indeed, a study from Amhara analyzed user preferences and uncovered that concrete slab platforms, which are considered as key components of hygienic pit latrines, were not preferred over dirt floors [68]. Unless the prevalent conception of hygienic toilets changes, health-related expectations will probably be not as effective for catalyzing the demand for upgrading sanitation facilities in Ethiopia as they have been for the initial adoption of basic latrines. Although other non-health motivations may be more influential, there has been very little research on the demand for latrine upgrading and its influencers in Ethiopia thus far.

### Limitations

The inclusion criteria were designed by considering our primary objective to review evidence on factors influencing household-level sanitation outcomes. Pooled averages of latrine coverage and use presented above thus did not cover findings from excluded studies. We covered research evidence solely from studies that analyzed sanitation outcomes at the household level. Studies that addressed sanitation in Ethiopia at other levels were not considered, though they may report findings on sanitation drivers which also impact household-level sanitation (e.g., ecological studies, policy analyses, implementation studies). The representation of outcomes, factors, and their relationships were assessed by counting their occurrences. Although we extracted information on the effect sizes (from quantitative studies), we did not use them for our synthesis due to the high between-study heterogeneity. In addition, we only extracted information on factors that were found significant but not on insignificant relationships. Almost all studies in our sample were observational studies that measured statistical associations and not cause-and-effect relationships. We tried to reflect specific qualitative explanations provided for the relationships in the analyzed studies. However, these interpretations of underlying mechanisms were sometimes ambiguous (as discussed above), speculative, and not always available.

### Conclusion

This review assessed available research on the drivers of household-level sanitation outcomes in Ethiopia. The findings may help practitioners to understand what the key types of sanitation drivers are in the Ethiopian context and how they are related to distinct sanitation outcomes at the micro-level. They may also inform researchers and policymakers about the nature of available evidence, gaps in it, and priority directions. Despite the reduction of OD in Ethiopia, hygienic sanitation is rather the exception than the rule across the country. We argued that the focus of research mainly emulated the focus of the approach chosen for national sanitation strategy (CLTS) on initial latrine adoption and use. Key areas such as a demand for upgrading sanitation facilities and a variety of issues on the supply side have been almost ignored in both policy and research. We also call for a more critical approach to sanitation research in Ethiopia.

## Supplementary Information

**Additional file 1: Table S1.** List of considered studies and their descriptive characteristics. **Table S2.** Extraction of measures of latrine coverage and use. **Table S3.** Links between types of outcomes and factors. **Table S4.** Number of identified relationships between factors (aggregated by THEMES) and sanitation outcomes (aggregated by TYPES). **Table S5.** Number of identified relationships between factors (aggregated by SUB-THEMES) and sanitation outcomes (aggregated by TYPES). **Table S6.** Sums of identified DIRECTIONAL relationships between factors (aggregated by SUB-THEMES) and sanitation outcomes (aggregated by TYPES).

## Data Availability

Data are available as supplementary materials submitted together with the manuscript.

## References

[1] UN (2015). Transforming our world: the 2030 agenda for sustainable development.

[2] Moyer JD, Hedden S (2020). Are we on the right path to achieve the sustainable development goals?. World Dev.

[3] WHO/UNICEF (2021). Progress on household drinking water, sanitation and hygiene 2000-2020: Five years into the SDGs.

[4] Garn JV, Sclar GD, Freeman MC, Penakalapati G, Alexander KT, Brooks P (2017). The impact of sanitation interventions on latrine coverage and latrine use: a systematic review and meta-analysis. Int J Hyg Environ Health.

[5] Sclar GD, Penakalapati G, Amato HK, Garn JV, Alexander K, Freeman MC (2016). Assessing the impact of sanitation on indicators of fecal exposure along principal transmission pathways: a systematic review. Int J Hyg Environ Health.

[6] De Buck E, Van Remoortel H, Hannes K, Govender T, Naidoo S, Avau B (2017). Approaches to promote handwashing and sanitation behaviour change in low-and middle-income countries: a mixed method systematic review. Campbell Syst Rev.

[7] Freeman MC, Garn JV, Sclar GD, Boisson S, Medlicott K, Alexander KT (2017). The impact of sanitation on infectious disease and nutritional status: a systematic review and meta-analysis. Int J Hyg Environ Health.

[8] Venkataramanan V, Crocker J, Karon A, Bartram J (2018). Community-led total sanitation: a mixed-methods systematic review of evidence and its quality. Environ Health Perspect.

[9] Chirgwin H, Cairncross S, Zehra D, Sharma Waddington H (2021). Interventions promoting uptake of water, sanitation and hygiene (WASH) technologies in low-and middle-income countries: an evidence and gap map of effectiveness studies. Campbell Syst Rev.

[10] Pfadenhauer LM, Gerhardus A, Mozygemba K, Lysdahl KB, Booth A, Hofmann B (2017). Making sense of complexity in context and implementation: the Context and Implementation of Complex Interventions (CICI) framework. Implement Sci.

[11] Winter S, Dreibelbis R, Barchi F (2018). Context matters: a multicountry analysis of individual-and neighbourhood-level factors associated with women’s sanitation use in sub-Saharan Africa. Tropical Med Int Health.

[12] Whittington D, Radin M, Jeuland M (2020). Evidence-based policy analysis? The strange case of the randomized controlled trials of community-led total sanitation. Oxf Rev Econ Policy.

[13] Chakraborty S, Novotný J, Das J, Bardhan A, Roy S, Mondal S (2022). Geography matters for sanitation! Spatial heterogeneity of the district-level correlates of open defecation in India. Singap J Trop Geogr.

[14] Dreibelbis R, Winch PJ, Leontsini E, Hulland KR, Ram PK, Unicomb L, Luby SP (2013). The integrated behavioural model for water, sanitation, and hygiene: a systematic review of behavioural models and a framework for designing and evaluating behaviour change interventions in infrastructure-restricted settings. BMC Public Health.

[15] Mosler HJ (2012). A systematic approach to behavior change interventions for the water and sanitation sector in developing countries: a conceptual model, a review, and a guideline. Int J Environ Health Res.

[16] WHO/UNICEF (2019). Progress on household drinking water, sanitation and hygiene 2000-2017: special focus on inequalities.

[17] MoH (2005). National hygiene and sanitation strategy for Ethiopia.

[18] MoH (2011). National hygiene & sanitation strategic action plan for rural, per-urban & informal settlements in Ethiopia.

[19] MoH (2013). Ethiopian national sanitation marketing guideline.

[20] MoH (2015). Health Sector Transformation Plan 2015/16-2019/20.

[21] Assefa Y, Gelaw YA, Hill PS, Taye BW, Van Damme W (2019). Community health extension program of Ethiopia, 2003–2018: successes and challenges toward universal coverage for primary healthcare services. Glob Health.

[22] Peal AJ, Evans BE, van der Voorden C (2010). Hygiene and sanitation software: an overview of approaches.

[23] Abebe TA, Tucho GT (2020). Open defecation-free slippage and its associated factors in Ethiopia: a systematic review. Syst Rev.

[24] OWNP (2019). Ethiopia One WASH National Program report.

[25] Bakker E, Feldman P (2021). Ethiopia’s business environment and how it influences WASH market development. Updated Ed.

[26] Novotný J, Hasman J, Lepič M (2018). Contextual factors and motivations affecting rural community sanitation in low-and middle-income countries: a systematic review. Int J Hyg Environ Health.

[27] Neyeloff JL, Fuchs SC, Moreira LB (2012). Meta-analyses and Forest plots using a Microsoft excel spreadsheet: step-by-step guide focusing on descriptive data analysis. BMC Res Notes.

[28] Shannon P, Markiel A, Ozier O, Baliga NS, Wang JT, Ramage D (2003). Cytoscape: a software environment for integrated models of biomolecular interaction networks. Genome Res.

[29] Abebe W, Earsido A, Taye S, Assefa M, Eyasu A, Godebo G (2018). Prevalence and antibiotic susceptibility patterns of Shigella and Salmonella among children aged below five years with Diarrhoea attending Nigist Eleni Mohammed memorial hospital, South Ethiopia. BMC Pediatr.

[30] Oswald WE, Stewart AE, Flanders WD, Kramer MR, Endeshaw T, Zerihun M (2016). Prediction of low community sanitation coverage using environmental and sociodemographic factors in Amhara Region, Ethiopia. Am J Trop Med Hyg.

[31] Shine S, Muhamud S, Adanew S, Demelash A, Abate M (2020). Prevalence and associated factors of diarrhea among under-five children in Debre Berhan town, Ethiopia 2018: a cross sectional study. BMC Infect Dis.

[32] O'Loughlin R, Fentie G, Flannery B, Emerson PM (2006). Follow-up of a low cost latrine promotion programme in one district of Amhara, Ethiopia: characteristics of early adopters and non-adopters. Tropical Med Int Health.

[33] Tessema RA (2017). Assessment of the implementation of community-led total sanitation, hygiene, and associated factors in Diretiyara district, Eastern Ethiopia. PLoS One.

[34] UNICEF (2016). Outcome evaluation of community-led total sanitation and hygiene: (CLTSH) program in Ethiopia from 2012-2015. BDS-Center for Development Research.

[35] Adank M, Butterworth J, Godfrey S, Abera M (2016). Looking beyond headline indicators: water and sanitation services in small towns in Ethiopia. J Water Sanit Hyg Dev.

[36] Novotný J, Humňalová H, Kolomazníková J (2018). The social and political construction of latrines in rural Ethiopia. J Rural Stud.

[37] Tulu L, Kumie A, Hawas SB, Demissie HF, Segni MT (2017). Latrine utilization and associated factors among kebeles implementing and non implementing urban community led total sanitation and hygiene in Hawassa town, Ethiopia. Afr J Environ Sci Technol.

[38] Abebe AM, Kassaw MW, Mekuria AD, Yehualshet SS, Fenta EA. Latrine utilization and associated factors in Mehal Meda town in North Shewa Zone, Amhara Region, Ethiopia, 2019. Biomed Res Int. 2020. 10.1155/2020/7310925. 10.1155/2020/7310925 PMC7321497 32685523

[39] Asnake D, Adane M (2020). Household latrine utilization and associated factors in semi-urban areas of northeastern Ethiopia. PLoS One.

[40] Alemu F, Kumie A, Medhin G, Gebre T, Godfrey P (2017). A socio-ecological analysis of barriers to the adoption, sustainability and consistent use of sanitation facilities in rural Ethiopia. BMC Public Health.

[41] Tamene A, Afework A (2021). Exploring barriers to the adoption and utilization of improved latrine facilities in rural Ethiopia: an integrated behavioral model for water, sanitation and hygiene (IBM-WASH) approach. PLoS One.

[42] Ngondi J, Teferi T, Gebre T, Shargie EB, Zerihun M, Ayele B (2010). Effect of a community intervention with pit latrines in five districts of Amhara, Ethiopia. Tropical Med Int Health.

[43] Crocker J, Geremew A, Atalie F, Yetie M, Bartram J (2016). Teachers and sanitation promotion: an assessment of community-led total sanitation in Ethiopia. Environ Sci Technol.

[44] Crocker J, Saywell D, Bartram J (2017). Sustainability of community-led total sanitation outcomes: evidence from Ethiopia and Ghana. Int J Hyg Environ Health.

[45] Ross RK, King JD, Damte M, Ayalew F, Gebre T, Cromwell EA (2011). Evaluation of household latrine coverage in Kewot woreda, Ethiopia, 3 years after implementing interventions to control blinding trachoma. Int Health.

[46] Fry D, Mideksa D, Ambelu A, Feyisa Y, Abaire B, Cunliffe K, Freeman MC (2015). Adoption and sustained use of the arborloo in rural Ethiopia: a cross-sectional study. J Water Sanit Hyg Dev.

[47] Aiemjoy K, Stoller NE, Gebresillasie S, Shiferaw A, Tadesse Z, Sewent T (2017). Is using a latrine “a strange thing to do”? A mixed-methods study of sanitation preference and behaviors in rural Ethiopia. Am J Trop Med Hyg.

[48] Novotný J, Kolomazníková J, Humňalová H (2017). The role of perceived social norms in rural sanitation: an explorative study from infrastructure-restricted settings of South Ethiopia. Int J Environ Res Public Health.

[49] Belachew AB, Abrha MB, Gebrezgi ZA, Tekle DY (2018). Availability and utilization of sanitation facilities in Enderta district, Tigray, Ethiopia. J Prev Med Hyg.

[50] Anteneh A, Kumie A (2010). Assessment of the impact of latrine utilization on diarrhoeal diseases in the rural community of Hulet Ejju Enessie Woreda, East Gojjam Zone. Amhara Region. Ethiop J Health Dev..

[51] Yimam YT, Gelaye KA, Chercos DH (2014). Latrine utilization and associated factors among people living in rural areas of Denbia district, Northwest Ethiopia, 2013, a cross-sectional study. Pan Afr Med J.

[52] Chanie T, Gedefaw M, Ketema K (2016). Latrine utilization and associated factors in rural community of Aneded district, North West Ethiopia, 2014. J Community Med Health Educ.

[53] Gebremedhin G, Tetemke D, Gebremedhin M, Kahsay G, Zelalem H, Syum H, Gerensea H (2018). Factors associated with latrine utilization among model and non-model families in Laelai Maichew Woreda, Aksum, Tigray, Ethiopia: comparative community based study. BMC Res Notes.

[54] Chambers KG, Carrico AR, Cook SM (2021). Drivers of sustained sanitation access: social network and demographic predictors of latrine reconstruction after flooding disasters. Environ Sci Water Res Technol.

[55] Ashenafi T, Dadi AF, Gizaw Z (2018). Latrine utilization and associated factors among Kebeles declared open defecation free in Wondo Genet district, South Ethiopia, 2015. ISABB J Health Environ Sci.

[56] Temesgen A, Molla Adane M, Birara A, Shibabaw T (2021). Having a latrine facility is not a guarantee for eliminating open defecation owing to socio-demographic and environmental factors: the case of Machakel district in Ethiopia. PLoS One.

[57] Gedefaw M, Amsalu Y, Tarekegn M, Awoke W (2015). Opportunities, and challenges of latrine utilization among rural communities of Awabel District, Northwest Ethiopia, 2014. Open J Epidemiol.

[58] Debesay N, Ingale L, Gebresilassie A, Assefa H, Yemane D (2010). Latrine utilization and associated factors in the rural communities of Gulomekada District, Tigray Region, North Ethiopia, 2013: a community based cross-sectional study. J Community Med Health Educ.

[59] Alemu F, Kumie A, Medhin G, Gasana J (2018). The role of psychological factors in predicting latrine ownership and consistent latrine use in rural Ethiopia: a cross-sectional study. BMC Public Health.

[60] Koyra HC, Sorato MM, Unasho YS, Kanche ZZ (2017). Latrine utilization and associated factors in rural Community of Chencha District, southern Ethiopia: a community based cross-sectional study. Am J Public Health Res.

[61] Anthonj C, Fleming L, Godfrey S, Ambelu A, Bevan J, Cronk R, Bartram J (2018). Health risk perceptions are associated with domestic use of basic water and sanitation services—evidence from rural Ethiopia. Int J Environ Res Public Health.

[62] Aragie S, Wittberg DM, Tadesse W, Dagnew A, Hailu D, Chernet A (2022). Water, sanitation, and hygiene for control of trachoma in Ethiopia (WUHA): a two-arm, parallel-group, cluster-randomised trial. Lancet Glob Health.

[63] Freeman MC, Delea MG, Snyder JS, Garn JV, Belew M, Caruso BA (2022). The impact of a demand-side sanitation and hygiene promotion intervention on sustained behavior change and health in Amhara, Ethiopia: a cluster-randomized trial. PLoS Glob Public Health.

[64] Jenkins MW, Freeman MC, Routray P (2014). Measuring the safety of excreta disposal behavior in India with the new safe san index: reliability, validity and utility. Int J Environ Res Public Health.

[65] Delea MG, Nagel CL, Thomas EA, Halder AK, Amin N, Shoab AK (2017). Comparison of respondent-reported and sensor-recorded latrine utilization measures in rural Bangladesh: a cross-sectional study. Trans R Soc Trop Med Hyg.

[66] Stoller NE, Gebre T, Ayele B, Zerihun M, Assefa Y, Habte D (2011). Efficacy of latrine promotion on emergence of infection with ocular Chlamydia trachomatis after mass antibiotic treatment: a cluster-randomized trial. Int Health.

[67] Oswald WE, Stewart AE, Kramer MR, Endeshaw T, Zerihun M, Melak B (2017). Active trachoma and community use of sanitation, Ethiopia. Bull World Health Organ.

[68] Goddard FG, Delea MG, Sclar GD, Woreta M, Zewudie K, Freeman MC (2018). Quantifying user preferences for sanitation construction and use: Application of discrete choice experiments in Amhara, Ethiopia. Tropical Med Int Health.

